# Differences in Health-related Quality of Life and Mental Health by Living Arrangement among Korean Elderly in the KNHANES 2010–2012

**Published:** 2017-11

**Authors:** Yeunhee KWAK, Haekyung CHUNG, Yoonjung KIM

**Affiliations:** Red Cross College of Nursing, Chung-Ang University, Seoul, Republic of Korea

**Keywords:** Quality of life, Mental health, Living arrangements, Elderly

## Abstract

**Background:**

This cross-sectional study examined the association between types of living arrangements, quality of life, and mental health of the Korean elderly.

**Methods:**

We used secondary data analysis from the data of 4248 elderly people aged 65 yr or older that completed the Korea National Health and Nutrition Examination Survey (2010–2012). Data concerning participants’ demographic characteristics, living arrangements, quality of life, and mental health were used. Data were analyzed using the SAS survey procedure.

**Results:**

The living arrangements were as follows: living alone=18.3%, living with a spouse only =44.5%, living with family without a spouse =13.4%, and living with family including a spouse=23.8%. Mobility, self-care, usual activity, pain/discomfort, and anxiety/depression significantly differed by living arrangement. In the final model corrected for covariance, for the elderly living with their families without a spouse compared to the elderly living with a spouse only, the odds ratios were the following: stress =1.40 (95% CI: 1.03–1.91), depression=1.48 (95% CI: 1.07–2.04), and suicidal ideation=1.48 (95% CI: 1.10–2.00). The odds ratio of suicidal ideation of elderly living alone compared to the elderly living with a spouse only was 1.32 (95% CI: 1.01–1.72). Finally, the elderly living with family without a spouse or living alone had an increased risk of stress, depression, and suicidal ideation. In addition, they had decreased health-related quality of life.

**Conclusion:**

Health-related quality of life and mental health differ by living arrangement in elderly adults. Therefore, interventions to improve quality of life and mental health for the elderly who are living without a spouse are necessary.

## Introduction

Of the Organization for Economic Co-operation and Development (OECD) member countries, Korea has the highest aging rate and is expected to become an “aged society” in 2017 and a “super-aged” society by 2026 (1). Such rapid aging of the Korean society aroused attention towards the problems of the elderly including quality of life (QoL) and health concerns. One of the most remarkable features of the health problems of an aging population is an increase in depression.

Depression symptoms occur in approximately 20%–50% of the elderly population (2). Additionally, the mortality rate due to suicide in Korea in 2011 was 33.3 per 100000 people, which was more than 2.6 times higher than the average suicide rate (12.6 per 100000) of other OECD member countries (3). By age, the suicide rate of the elderly aged 80 yr or older (123.3 per 100000) was approximately 5 times higher than those aged in their 20s (24.4 per 100000) and 30s (29.6 per 100000) (4), indicating that elderly suicide is very serious. Therefore, it is necessary to identify the elderly’s mental health concerns, including low QoL, depression, and suicidal ideation.

The elderly’s life satisfaction depends on their family relationships because family helps provide important psychological and social support (5). Consequently, living with a family becomes an environmental factor that determines the elderly’s QoL. Because it acts as a support system to help adapt to a crisis, living with a family can provide energy, relieve stress, and reduce the risk of disease (6). Although the proportion of older adults is increasing with the increase in life expectancy, the number of elderly individuals living alone has also increased, as it is becoming less culturally acceptable to live with one’s children (7).

The elderly that are living with their children can receive economic and social support from their children; however, single senior households are limited economically and elders are more likely to have various negative health behaviors due to a lack of social support provided by family members (8, 9). In addition, elderly living with a spouse may feel less socially isolated because relying on another might diminish anxieties, obsessive-compulsive neurosis, and depression severity (8, 10). For elderly living alone, reduction of familial support may cause mental health problems such as increased depression and suicidal ideation (11–13). Several other studies have revealed differences in the QoL and mental health of elderly individuals who are living alone or living with others (12, 14, 15). However, most studies have only compared either a) living alone and not living alone or b) living with a spouse or living without a spouse; moreover, few studies have specifically analyzed health-related QoL and mental health per living arrangement. In addition, the association between health-related QoL, mental health, and living arrangement has not been studied in a large elderly population independent of potential confounding factors. Therefore, this study was conducted to identify the effect of living arrangement on the QoL and mental health of the elderly by using raw data from the Korea National Health and Nutrition Examination Survey (KNHANES V) (2010–2012), which was a large-scale survey with representativeness and reliability.

The specific objectives of this study were as follows: 1) to identify the socio-demographic characteristics according to the elderly’s living arrangement; 2) to examine differences in varied areas of elderly adult’s health-related QoL according to living arrangement; and 3) to identify the association between elderly adult’s health-related QoL, mental health, and living arrangements.

## Materials and Methods

### Study population

The KNHANES V (2010–2012) was conducted by the Korea Centers for Disease Control and Prevention (KCDC) to identify the health and nutritional state of Koreans since 1998.

It was conducted with approval from the KCDC’s research Ethics Committee (IRB No. 2012-01EXP-01-2C).

**Fig. 1:**
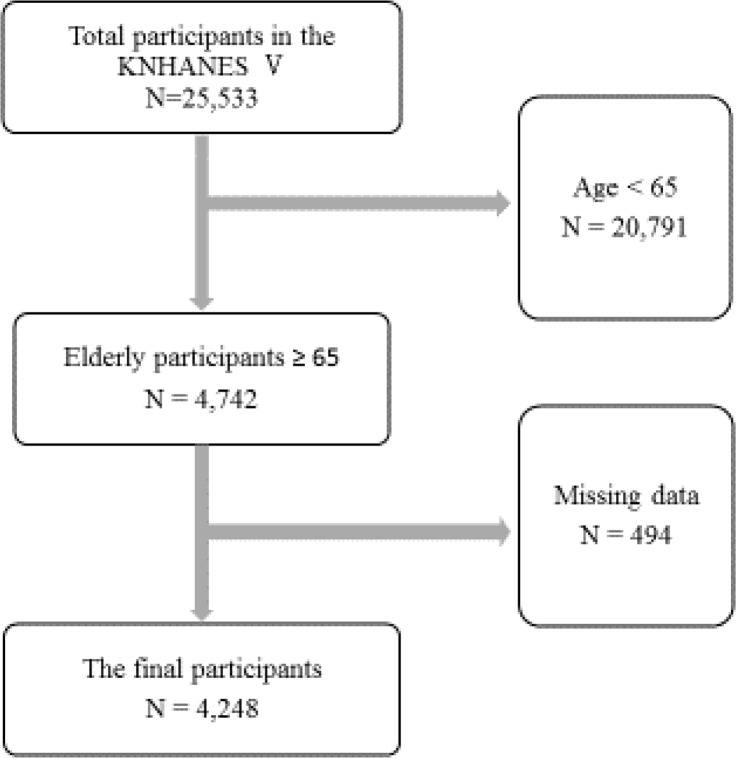
Flow diagrams for the selection of the study population

This cross-sectional study was conducted as a secondary analysis by using the raw data from KNHANES V (2010–2012) under formal approval of the appropriate agency. The KNHANES V (2010–2012) was a nationally representative, cross-sectional survey that targeted non-institutionalized Korean people. Samples were extracted to represent the Korean population by using stratified, multistaged, clustered, and probability design. In addition, by introducing the rolling survey sampling method, the rolling samples of each survey year became the probability samples representing the country and were independent and homogeneous characteristics. Sampling weights indicating the probability of being sampled were assigned to each participant, thus producing results that represented the entire Korean population. Selected participants were sent a notice of selection prior to survey commencement, identities were verified, the study’s purpose was explained, and written informed consent was obtained. A week prior to survey commencement, participants attended an appointment to complete health and nutrition surveys and undergo a clinical examination. The health and nutrition surveys included one-on-one interviews and self-report questionnaires, while the clinical examination was conducted by a specialized examination team at the KCDC including nurses, physicians, nutritionists, and health science majors. After being selected and finishing their training and practice (2–4 wk), team members were sent to the research field. Their research performance ability was continuously verified through regular education (7 times per year) and on-site quality management. The KCDC, through its sub-department and mediation consult committee, oversaw the quality of the KNHANES data collected (16). Of the 10938 survey participants, analyzed participants included 8958 people (participation rate=81.9%) in the first year (2010) (16). Of the 10589 survey participants, analyzed participants included 8.518 people (participation rate=80.4%) in the second year (2011) (17). Of the 10589 survey participants, analyzed participants included 8057 people (participation rate=80.0%) in the third year (2012) (18). This study analyzed elderly adults aged 65 yr or older (N=4742) using the KNHANES V (2010–2012) data (N=25533). Four-hundred ninety-four people were excluded for missing questionnaire data leaving 4248 individuals for study analysis (Fig. 1).

### Measures

#### Health-related QoL

The 5-dimension European Quality of Life Questionnaire (EQ-5D) (19) was employed to measure health-related QoL. The EQ-5D was developed to measure simple and overall health for clinical and economic evaluation. It comprises five dimensions: mobility, self-care, usual activities, pain/discomfort, and anxiety/depression. Each dimension can be answered with three responses: “*no problems*,” “*some problems*,” or “*severe problems*.” Health-related QoL scores were obtained by applying a weighted value to each measured value of questions about these five dimensions and the range of the values was distributed between 1 point (meaning a completely healthy state) and -1 point, meaning a health state was reported as “worse than death” (20).

#### Mental health

To assess participants’ mental health, we used to stress, depression, and suicidal ideation. For stress, the responses of “I feel very much” and “I feel much” to the question, “How much stress do you feel during normal everyday life?” were classified into “yes” and the answers of “I feel a little bit” and “I hardly feel any” were categorized as “no” (21). For depression, answers to the question, “Have you ever felt sad or depressed enough that it interfered with your daily life continuously for more than 2 wk during the past year?” were classified as either “yes” or “no” (22, 23). For suicidal ideation, answers to the question, “Did you ever think that you wanted to die during the past year?” were categorized as either “yes” or “no” (24).

#### Living arrangement type

Participants’ living arrangements were classified into four types: (1) living alone, (2) living with a spouse only, (3) living with family without a spouse, and (4) living with family including a spouse; living alone included being single, divorced, or widowed.

#### Other study variables

Age, sex, living location (urban or rural), education, economic status, smoking, and drinking were examined as demographic variables. Education was classified as elementary or lower, middle school graduate, and high school graduate or higher. Economic status was determined as monthly household income divided by the square root of the number of household members. For smoking, smoked more than 100 cigarettes during one’s lifetime and currently, smoking was classified as “yes” and other cases were classified as “no” (25, 26). Drinking at least once a month during the last year was classified as “yes” and other cases were classified as “no.”

#### Statistical analysis

All data were presented as mean ± SE for continuous variables or as n (%) for categorical variables. The SAS survey procedure (ver. 9.3; SAS Institute Inc., Cary NC, USA) was used to run a complex sample design based on data analysis from the survey data; this provided sampling weights of KNHANES V (2010–2012) and nationally representative estimates. The significance threshold was .05. The difference in living arrangements for the demographic characteristics of participants was tested using a *t*-test and a χ^2^ test. For the difference in each dimension of EQ-5D according to living arrangement, a χ^2^ test was used. To identify the association between the EQ-5D index, health-related QoL, and participants’ living arrangements, the participants’ demographic characteristics were controlled for and an analysis of covariance was conducted. Finally, to determine the association between living arrangement and mental health, a logistic regression analysis was conducted, adjusted for the participants’ demographic characteristics.

## Results

### Sociodemographic characteristics of the study population

Participants’ sociodemographic characteristics according to living arrangement are presented in Table 1. In the study population, living with a spouse only accounted for the largest proportion. The average age of the elderly living alone was the highest. The largest proportion of women were living alone or living with family without a spouse. For education, elementary school graduation or lower was the most common in all four types of living arrangement and the most common for elderly living alone. The proportion of very low economic status was highest in elderly living alone, living with a spouse only, and living with family without a spouse, respectively. The proportion of low economic status was highest in elderly living with family including a spouse. All four types of living arrangements were present in cities and the rate of currently not smoking and drinking were the most common.

**Table 1:** Participants’ sociodemographic characteristics by living arrangements (N = 4248)

***Characteristic***	***Living arrangements***				***P-value***
	***Living alone***	***Living with a spouse only***	***Living with family (without a spouse)***	***Living with family (with a spouse)***	
	***(n = 776)***	***(n = 1,890)***	***(n = 571)***	***(n = 1,011)***	
	Mean ± SE or % (SE)	Mean ± SE or % (SE)	Mean ± SE or % (SE)	Mean ± SE or % (SE)	
Age (years)	74.2 ± 0.2	71.9 ± 0.1	73.3 ± 0.2	72.1 ± 0.2	< .001
Sex					< .001
Male	16.9 (1.3)	57.8 (1.1)	22.1 (1.7)	47.7 (1.6)	
Female	83.1 (1.3)	42.2 (1.1)	77.9 (1.7)	52.3 (1.6)	
Education					< .001
≤ Elementary school	82.5 (1.4)	58.3 (1.1)	75.4 (1.8)	63.0 (1.5)	
Middle school	7.9 (1.0)	15.1 (0.8)	8.4 (1.2)	12.3 (1.0)	
≥ High school	9.7 (1.1)	26.6 (1.0)	16.1 (1.5)	24.7 (1.4)	
Economic status					< .001
Very low	80.1 (1.4)	57.2 (1.1)	40.1 (2.1)	24.9 (1.4)	
Low	13.5 (1.2)	27.3 (1.0)	26.8 (1.9)	29.4 (1.5)	
High	4.4 (0.7)	9.5 (0.7)	19.3 (1.7)	24.4 (1.4)	
Very high	1.9 (0.5)	6.1 (0.6)	13.8 (1.5)	21.3 (1.3)	
Living place					< .001
Urban	61.7 (1.7)	61.2 (1.1)	75.5 (1.8)	78.6 (1.3)	
Rural	38.3 (1.7)	38.8 (1.1)	24.5 (1.8)	21.4 (1.3)	
Smoking (current)					.002
No	88.5 (1.1)	85.8 (0.8)	91.6 (1.2)	86.5 (1.1)	
Yes	11.5 (1.1)	14.2 (0.8)	8.4 (1.2)	13.5 (1.1)	
Drinking (current)					< .001
No	77.2 (1.5)	59.2 (1.1)	73.7 (1.8)	63.7 (1.5)	
Yes	22.8 (1.5)	40.8 (1.1)	26.3 (1.8)	36.3 (1.5)	

Note: SE = standard error. The statistical differences were analyzed using a *t*-test and a χ^2^ test.

### Association between elderly adult’s health-related QoL, mental health, and living arrangements

The differences in living arrangements and each EQ-5D dimensions are shown in Table 2. Each dimension of EQ-5D significantly differed by living arrangement. The percentage with a problem in mobility in the elderly living alone (54.4%) was the highest and the percentage with a problem in the elderly living alone and the elderly living with family without a spouse was relatively higher in self-care, usual activity, pain/discomfort, and anxiety/depression. Table 3 shows the association between health-related QoL (EQ-5D index) and mental health (stress, depression, and suicidal ideation) according to participants’ living arrangement. Model 1 has no variables adjusted. In Model 2, age and sex were adjusted for. In Model 3, education, economic status, living place, smoking, and drinking were additionally adjusted. Model 1 and 2 showed statistical differences in the EQ-5D index according to elderly’s living arrangement. However, the EQ-5D index did not significantly differ in Model 3, where all demographic variables were adjusted. When comparing mental health regarding couple-living arrangements, stress showed a significant difference in Model 1 and Model 3. Furthermore, depression and suicidal ideation showed a significant difference in all three Models.

**Table 2:** Differences in EQ-5D and living arrangements (N=4248)

***EQ-5D***	***Living arrangements***				***P-value***
	***Living alone***	***Living with a spouse only***	***Living with family (without a spouse)***	***Living with family (with a spouse)***	
	***n (%)***	***n (%)***	***n (%)***	***n (%)***	
Mobility					< .001
No problem	354 (45.6)	1,169 (61.9)	307 (53.8)	636 (62.9)	
Some problem	390 (50.3)	681 (36.0)	243 (42.5)	360 (35.6)	
Severe problem	32 (4.1)	40 (2.1)	21 (3.7)	15 (1.5)	
Self-care					< .001
No problem	625 (80.5)	1,650 (87.3)	471 (82.5)	898 (88.8)	
Some problem	139 (17.9)	222 (11.7)	89 (16.6)	102 (10.1)	
Severe problem	12 (1.6)	18 (1.0)	11 (1.9)	11 (1.1)	
Usual activity					< .001
No problem	495 (63.8)	1,422 (75.2)	392 (68.7)	777 (76.8)	
Some problem	225 (29.0)	413 (21.9)	147 (25.7)	203 (20.1)	
Severe problem	56 (7.2)	55 (2.9)	32 (5.6)	31 (1.1)	
Pain/discomfort					< .001
No problem	392 (50.5)	1,177 (62.3)	304 (53.3)	643 (63.6)	
Some problem	283 (36.5)	582 (30.8)	208 (36.4)	309 (30.6)	
Severe problem	101 (13.0)	131 (6.9)	59 (10.3)	59 (5.8)	
Anxiety/depression					< .001
No problem	603 (77.7)	1,582 (83.7)	455 (79.7)	870 (86.0)	
Some problem	150 (19.3)	281 (14.9)	101 (17.7)	124 (12.3)	
Severe problem	23 (3.0)	27 (1.4)	15 (2.6)	17 (1.7)	

Note: EQ-5D= The 5-dimension European Quality of Life Questionnaire. The statistical differences were analyzed using a χ^2^ test.

**Table 3:** Health-related quality of life and mental health according to living arrangements

		***EQ-5D index***	***Stress***	***Depression***	***Suicidal ideation***
		***Mean ± SE***	***OR (95% CI)***	***OR (95% CI)***	***OR (95% CI)***
Model 1					
Living arrangements	Living alone	0.80 ± 0.01	1.41 (1.10, 1.81)	1.81 (1.37, 2.39)	2.00 (1.57, 2.56)
	Living with a spouse only	0.87 ± 0.01	1	1	1
	Living with family (without a spouse)	0.82 ± 0.01	1.77 (1.34, 2.33)	1.70 (1.27, 2.28)	1.81(1.38, 2.39)
	Living with family (with a spouse)	0.87 ± 0.01	1.16 (0.92, 1.46)	1.13 (0.84, 1.51)	1.17 (0.92, 1.49)
	P-value	< .001			
Model 2					
Living arrangements	Living alone	0.83 ± 0.01	1.01 (0.77, 1.32)	1.42 (1.05, 1.92)	1.46 (1.12, 1.90)
	Living with a spouse only	0.86 ± 0.01	1	1	1
	Living with family (without a spouse)	0.84 ± 0.01	1.26 (0.93, 1.70)	1.35 (0.99, 1.84)	1.39 (1.04, 1.84)
	Living with family (with a spouse)	0.87 ± 0.01	1.06 (0.83, 1.35)	1.05 (0.79, 1.40)	1.06 (0.83, 1.36)
	P-value	< .001			
Model 3					
Living arrangements	Living alone	0.87 ± 0.01	0.93 (0.71, 1.22)	1.30 (0.96, 1.75)	1.32 (1.01, 1.72)
	Living with a spouse only	0.88 ± 0.01	1	1	1
	Living with family (without a spouse)	0.86 ± 0.01	1.40 (1.03, 1.91)	1.48 (1.07, 2.04)	1.48 (1.10, 2.00)
	Living with family (with a spouse)	0.88 ± 0.01	1.29 (0.98, 1.69)	1.25 (0.91, 1.72)	1.26 (0.97, 1.63)
	P-value	.071			

Note: OR = odds ratio; CI = confidence interval; EQ-5D = The 5-dimension European Quality of Life Questionnaire. Model 1 was not adjusted; Model 2 was adjusted for age and sex; Model 3 was the result of adjusted education, economic status, living place, smoking, and drinking from Model 2. The statistical methods were analyzed using an analysis of covariance and logistic regression analysis.

Therefore, in the final model with all covariates adjusted for, elderly living with family without a spouse had an increased risk of stress, depression, and suicidal ideation when compared with those living with a spouse only. Finally, elderly living alone showed an increased risk of suicidal ideation compared to elderly living with a spouse only.

## Discussion

In this study, the basic data for qualitative improvement of the elderly’s mental health and health-related QoL was considered by identifying their association with living arrangement. Living with a spouse only accounted for the largest proportion and the proportion of elderly living alone was 18.3%. This proportion is expected to increase to 21.6% in 2020 (1). This would be a further move away from the traditional family type that supports elderly parents. One possible reason for this “family breakdown” phenomenon is the increased participation of women in economic activities caused by industrialization. This is influenced by the improved education system, an increase in the divorce rate, nuclearization, and so on (27). Elderly adults living with their children receive economic and social support from their children; however, elderly living alone lack this support and face dire health circumstances (8, 28). A noticeable result in this study was the difference in sociodemographic characteristics according to participants’ living arrangement. Specifically, elderly adults living alone were very old, most were women, had a lower economic status, and they tended to have an education level of only elementary school or less. The education level and average monthly total income of elderly living alone were lower than those living with a spouse were (28, 29). In Korea, the elderly poverty rate in 2013 was 48.0%, which was 3.5 times higher than the overall poverty rate (13.7%). Moreover, older men and women had a poverty rate of 40.1% and 45.9%, respectively (30). Women account for more than two-thirds of the total elderly poverty population in Western industrial society (31), indicating that the elderly poverty problem is more severe in women. The poverty risk of older women was caused by the risk of poverty accumulated through their lifetime and having less advantageous income opportunities than men do (29). Therefore, it is necessary to identify the socio-demographic characteristics and living arrangements of the Korean elderly to help provide appropriate national policies, economic support, and social support.

Finally, there was no significant association between each EQ-5D dimension and living arrangement type in the final model; however, the health-related QoL of elderly living alone or living with family without a spouse was degraded. Consequently, elderly living alone had a higher rate of chronic disease than did elderly living with others (8), and no spousal results for signs of anxiety or obsessive-compulsive syndrome of living alone and depression severity were added (8, 10).

Elderly individuals living without a spouse had a higher risk of stress, depression, and suicidal ideation than did those who lived with a spouse. Moreover, elderly adults living alone had a higher risk of suicidal ideation compared to elderly who were living with a spouse only. In general, elderly Koreans living alone have been shown to be more to think of suicide and suffer depression than the elderly who are living with a spouse (28, 32). The prevalence of depressive symptoms was associated with the living arrangements of elderly Koreans: living alone was most strongly associated with depressive symptoms in elderly women and men (14). This was consistent with a previous study that found that Hispanics living alone report significantly higher levels of depression relative to Hispanics living with their spouse/partner (15). Additionally, social isolation or social relationships, lower social support in the elderly (33, 34), physical illness, and depression (35, 36) are associated with suicidal ideation. Elderly living alone or with family with a lack of social support without a spouse has a higher risk of depression and suicidal ideation, which supports the results of this study. Elderly adults living alone have been regarded as vulnerable targets and seen as having decreased quality of life or impaired mental health for some time; therefore, it is necessary to expand mental health services to include them and develop programs for systematic and discriminatory mental health promotion for elderly adults living without a spouse.

Living with a spouse positively affects health behaviors more so than does living with children. A possible reason for this effect is that spouses encourage each other to visit medical institutions for influenza vaccinations, cancer screening, suppression of smoking, and prevention of non-medical treatment (8). Moreover, elderly adults with spouse-centered support also have higher life satisfaction than do those with children-centered support in family relationships (7, 28). Therefore, multilateral efforts to reduce elderly divorce and for the couple to live a healthy life together are useful. In addition, there is a need for intervention measures and institutional strategies for improving the mental health of the elderly living without a spouse.

The major strength of this study is its large and nationally representative sample, which enabled this study to provide the primary evidence of how health-related QoL and mental health in elderly adults differ according to various living arrangements. This study may help healthcare providers understand the association between health-related QoL, mental health, and living arrangement type of community-dwelling elderly adults. Based on the results of this study, it is necessary to pay attention not only to the elderly living alone but also to the elderly living with their family without a spouse, to improve their mental health.

This study was limited by its cross-sectional design. Further longitudinal studies are necessary to determine causal relationships. Another limitation is that it used a measurement tool that assessed mental health by self-report. Since responses depended on respondents’ recall, this method may have been affected by recall bias. Third, mental health was assessed by using only one question to obtain limited answers. There might be some bias in the measurements of these variables.

## Conclusion

Health-related QoL and mental health of elderly Korean individuals differ according to their living arrangement. Those living alone and living with family without a spouse have an increased risk of stress, depression, and suicidal ideation. Therefore, early diagnosis and economic and social support are necessary to help prevent these outcomes.

## Ethical considerations

Ethical issues regarding plagiarism, informed consent, misconduct, data fabrication and/or falsification, double publication and/or submission, and redundancy have been observed by the author.
